# Aerobic exercise training improves hepatic and muscle insulin sensitivity, but reduces splanchnic glucose uptake in obese humans with type 2 diabetes

**DOI:** 10.1038/s41387-019-0090-0

**Published:** 2019-09-02

**Authors:** Justin M. Gregory, James A. Muldowney, Brian G. Engelhardt, Regina Tyree, Pam Marks-Shulman, Heidi J. Silver, E. Patrick Donahue, Dale S. Edgerton, Jason J. Winnick

**Affiliations:** 1 Ian M. Burr Division of Pediatric Endocrinology and Diabetes, Vanderbilt University School of Medicine, 1500 21st Ave, Suite 1514, Nashville, TN 37212 USA; 2 Division of Cardiovascular Medicine, Vanderbilt University School of Medicine, 2215 Garland Avenue, Nashville, TN 37232-6015 USA; 3 Division of Hematology and Oncology, Vanderbilt University School of Medicine, 2215 Garland Avenue, Nashville, TN 37232-6015 USA; 4 Center for Human Nutrition, Vanderbilt University School of Medicine, 2215 Garland Avenue, Nashville, TN 37232-6015 USA; 5 Section of Surgical Sciences, Vanderbilt University School of Medicine, 2215 Garland Avenue, Nashville, TN 37232-6015 USA; 6 Department of Molecular Physiology and Biophysics, Vanderbilt University School of Medicine, 2215 Garland Avenue, Nashville, TN 37232-6015 USA; 7 Division of Endocrinology, Diabetes and Metabolism, Department of Internal Medicine, University of Cincinnati College of Medicine, 231 Albert Sabin Way, Cincinnati, OH 45267-0547 USA

**Keywords:** Type 2 diabetes, Type 2 diabetes

## Abstract

**Background:**

Aerobic exercise training is known to have beneficial effects on whole-body glucose metabolism in people with type 2 diabetes (T2D). The responses of the liver to such training are less well understood. The purpose of this study was to determine the effect of aerobic exercise training on splanchnic glucose uptake (SGU) and insulin-mediated suppression of endogenous glucose production (EGP) in obese subjects with T2D.

**Methods:**

Participants included 11 obese humans with T2D, who underwent 15 ± 2 weeks of aerobic exercise training (AEX; *n* = 6) or remained sedentary for 15 ± 1 weeks (SED; *n* = 5). After an initial screening visit, each subject underwent an oral glucose load clamp and an isoglycemic/two-step (20 and 40 mU/m^2^/min) hyperinsulinemic clamp (ISO-clamp) to assess SGU and insulin-mediated suppression of EGP, respectively. After the intervention period, both tests were repeated.

**Results:**

In AEX, the ability of insulin to suppress EGP was improved during both the low (69 ± 9 and 80 ± 6% suppression; pre-post, respectively; *p* < 0.05) and high (67 ± 6 and 82 ± 4% suppression, respectively; *p* < 0.05) insulin infusion periods. Despite markedly improved muscle insulin sensitivity, SGU was reduced in AEX after training (22.9 ± 3.3 and 9.1 ± 6.0 g pre-post in AEX, respectively; *p* < 0.05).

**Conclusions:**

In obese T2D subjects, exercise training improves whole-body glucose metabolism, in part, by improving insulin-mediated suppression of EGP and enhancing muscle glucose uptake, which occur despite reduced SGU during an oral glucose challenge.

## Introduction

Type 2 diabetes (T2D) is a metabolic disease characterized by the dysfunction of several key glucoregulatory organs during the fasted state and in response to glucose ingestion^1^. Among these organs, impaired glucose metabolism by the liver is recognized as an important contributor to T2D because of the central role it plays in the regulation of both fasting glucose levels and glucose tolerance. In healthy humans, insulin and glucagon regulate hepatic glucose production (HGP) to maintain euglycemia during fasting. In contrast, hepatic insulin resistance, along with hyperglucagonemia^2^, increase fasting HGP in patients with T2D, thereby contributing to hyperglycemia^3^. Insulin signaling is also important for normal liver function during the postprandial state because it promotes hepatic glucose uptake and glycogen deposition, a complex process that accounts for the disposal of one-third of ingested carbohydrate^4–9^. It is therefore not surprising that T2D patients also exhibit impaired liver glucose uptake and glycogen synthesis during the postprandial state^5,10–15^.

Although treatments such as surgical weight loss^16^ and insulin-sensitizing medications^7,8^ can improve whole-body glucose metabolism in T2D patients, lifestyle intervention is the ideal method for improving glucoregulation in this population. Whereas the benefit of aerobic exercise training on skeletal muscle insulin sensitivity continues to be extensively investigated^17^, the effect of training on hepatic glucose metabolism is less clear. It has been shown previously that aerobic exercise training periods of ~12–16 weeks can enhance hepatic insulin sensitivity, manifest by improved suppression of HGP in response to submaximal doses of insulin^18,19^. However, it remains unclear whether these training-induced improvements in hepatic insulin sensitivity extend to enhanced hepatic glucose metabolism during the postprandial state. To this end, the purpose of this study was to determine how aerobic exercise training by subjects with T2D affects hepatic glucose metabolism during the fasting and postprandial states.

## Materials and methods

### Subjects

Vanderbilt University’s Institutional Review Board approved the methods of this study. Prior to enrollment, all subjects were informed of the risks associated with participation and provided written consent. Inclusion criteria included males and non-pregnant females aged 40–60 years with T2D, a BMI range of 30–40 kg/m^2^, a hemoglobin A1C of ≤8.5% and sedentary for the previous six months. Exclusion criteria included an abnormal exercise stress test and the presence of neuropathy, nephropathy, or retinopathy. Individuals reporting musculoskeletal or other conditions that would make strenuous exercise difficult or dangerous were also excluded.

### Study overview

For 5 days prior to each study visit, subjects discontinued their diabetes-related medications to minimize the confounding effect of the drugs on exercise-induced responses. Because of this, individuals taking insulin or thiazoladinediones (because of the long half-life) were excluded. Each subject underwent an initial screening visit during which baseline blood was drawn, followed by a 75 g oral glucose tolerance test (OGTT). After the OGTT, each subject performed a VO_2_ max test so that exercise prescriptions could be generated. At least 1 week after this screening visit, we assessed hepatic glucose metabolism using two protocols separated by at least one week. First, baseline splanchnic glucose uptake (SGU) was determined using the 75 g oral glucose load clamp technique (OGL-clamp). Second, hepatic insulin sensitivity was measured using the isoglycemic/ hyperinsulinemic clamp method (ISO-clamp). After the completion of these metabolic tests, subjects either remained sedentary (SED; *n* = 5) or participated in ~15 weeks of aerobic exercise training (AEX; *n* = 6). After the intervention period, each subject repeated the OGL-clamp and ISO-clamps in the same order. All subjects were instructed either to continue to exercise (AEX) or remain sedentary (SED) until the day before each post-intervention metabolic study.

### Screening visit

Each participant reported to the Vanderbilt Clinical Research Center (CRC) in the morning after an overnight fast. Upon arrival, an intravenous (IV) catheter was inserted into the antecubital vein and blood was drawn to measure hemoglobin A1C and a complete blood count. The following tests were then conducted:

### Oral glucose tolerance test

While resting comfortably in a hospital bed, blood samples were taken at min 0 for the measurement of basal plasma glucose and insulin. Next, each subject consumed a 75-g glucose solution within 5 min, after which plasma glucose and insulin levels were assessed for 2 h. A plasma glucose concentration >199 mg/dL after 2 h confirmed T2D.

### VO_2_ max/ EKG testing

After completing the OGTT, each subject underwent a VO_2_ max test, which included 12-lead EKG monitoring to screen for cardiac disease. A modified Balke 3.0 protocol was used, with each stage of the test lasting 3 min to allow oxygen consumption to reach a steady state. VO_2_ max was considered to be attained if two of the following three criteria were achieved; (1) a respiratory exchange ratio >1.10; (2) a heart rate within 10 beats of the age-predicted maximum (220-age); or (3) no change in oxygen consumption despite an increase in workload. Exercise prescriptions for the AEX group equaled the treadmill workload that solicited 70% of the individually measured VO_2_ max^20^.

### OGL-clamp

Splanchnic glucose uptake (SGU) was assessed using the OGL-clamp technique as previously described^5–7,9^. In brief, subjects discontinued their diabetes-related medications for 5 days prior to reporting to the CRC the evening before the metabolic study. Upon admission, each subject consumed a standardized meal and remained fasted thereafter. At ~7 a.m., one infusion catheter was inserted into one antecubital vein for infusions and another was inserted into the contralateral antecubital vein for periodic blood draws. A heating blanket was used to allow the draw of arterialized blood.

The OGL-clamp lasted 390 min and was divided into a 180-min euglycemic/ hyperinsulinemic lead-in period (−180-0 min), followed by the ingestion of a 75 g OGL and periodic blood draws to assess SGU (0–210 min). At −180 min, a primed (25 µCi), continuous (0.25 µCi/min) infusion of 3-^3^H glucose was begun, thereby allowing the assessment of glucose turnover. At the same time, a primed^21^, continuous (120 mU/m^2^/min) infusion of insulin was started to suppress HGP, while simultaneously stimulating muscle glucose uptake. During the first 3 h of the OGL-clamp (min −180 to 0), the plasma glucose level was measured every 5–10 min and euglycemia (~95 mg/dL) was maintained by a variable IV-infusion of 20% dextrose (d20) as necessary. During the final 30 min of this period, glucose turnover was measured to confirm that HGP was suppressed prior to the OGL.

At minute 0, while the IV-infusion of insulin was continued, each participant consumed a 75-g OGL, labeled with 500 mg acetaminophen, within 5 min. To minimize any rise in plasma glucose that would occur after the ingestion of the OGL, the pre-existing exogenous glucose infusion rate (GIR) was lowered as needed for each subject. The complete absorption of the OGL was indicated by a return of the exogenous GIR to a rate similar to what existed prior to the OGL. In total, 210 min after consumption of the OGL, the insulin infusion was discontinued and subjects were fed a meal. After stabilization of the plasma glucose level, the d20 infusion was stopped and the subject was discharged from the CRC, after which they resumed their previously existing medication regimen.

### ISO-clamp

Approximately 1 week after the OGL-clamp, hepatic insulin sensitivity was measured using the isoglycemic/ hyperinsulinemic clamp technique. For this visit, subjects again discontinued their diabetes-related medications 5 days prior. Upon reporting to the CRC, each subject ate dinner, then remained fasted for ~12 h.

The next morning, catheters were inserted and 3-^3^H glucose tracer was infused as described previously for the OGL-clamp. The initial 120 min of the study allowed for tracer equilibration, followed by a 30-min control period during which hormones, substrates, and glucose turnover rates were assessed during the basal state. The average plasma glucose level during this sampling period then became the isoglycemic level at which the subject’s plasma glucose would be clamped for the remainder of the study.

At minute 150, a primed^21^, continuous (20 mU/m^2^/min) infusion of insulin was started and maintained for the next 150 min (i.e., min 150–300). Isoglycemia was sustained during this period by monitoring the plasma glucose level every 5–10 min and infusing d20 as necessary. The tracer infusion rate from the stock solution was lowered from 0.25 to 0.06 µCi/min and the d20 infusate was labeled with 3-^3^H glucose as described previously^20^. As was the case with the basal period, hormones, substrates and glucose turnover were assessed during the final 30 min of this period.

During the final 150 min period (i.e., min 300–450), the insulin dose was increased to 40 mU/m^2^/min. Isoglycemia was maintained during this period by monitoring the plasma glucose level every 5–10 min and adjusting the d20 infusion rate as necessary. As was the case during the previous two periods, hormones, substrates, and glucose turnover were assessed during the final 30 min of this period. At min 450, the insulin and tracer infusions were discontinued and subjects were fed. After the plasma glucose level was stabilized, the exogenous glucose infusion was stopped and the subject was discharged and instructed to resume their medication regimen.

### Exercise training

Each subject assigned to the AEX group performed treadmill walking 4–5 days per week for ~15 weeks. Each session consisted of 2 × 25 min bouts of treadmill walking at 70% VO_2_ max. A 10-min break was allowed between bouts. Each session included a brief warm up and cool down period. Each subject in AEX was instructed to continue exercising until after the second ISO-Clamp (i.e., the second of the two clamp studies).

### Analysis of plasma hormones and metabolites

Plasma insulin, glucagon, cortisol, and c-peptide (Millipore, St. Louis, USA) were measured by the Vanderbilt Diabetes Research and Training Center’s (DRTC) hormone assay and analytical services core. Plasma glucose was measured using the glucose oxidase method (ref. ^22^; Beckman Instruments). Glycerol and lactate^23^ and plasma specific activity^20^ were measured as described previously and free fatty acids were measured using a commercially available kit (NEFA-HR kit; Wako Chemicals; Osaka, Japan).

### Calculations

Samples were taken every 10 min during the final 30 min of each clamp period (i.e. the euglycemic lead-in period of the OGL-clamp and the basal, low- and high-insulin periods of the isoglycemic clamp) and analyzed for glucose specific activity at each time point. During the basal state of the ISO-clamp, glucose turnover was calculated for each time point by dividing the tracer infusion rate by the glucose specific activity. During the lead-in period of the OGL-clamp and the latter two periods of the ISO-clamp, when exogenous glucose was being infused, the total rate of appearance (Ra) of glucose was calculated for each time point the same way, whereas endogenous glucose production (EGP) was calculated by subtracting the exogenous GIR from Ra.

Splanchnic glucose uptake (SGU) was calculated by subtracting the amount of glucose escaping the splanchnic bed from the total amount of glucose ingested (75 g). Splanchnic glucose escape (SGE) can be determined by calculating the reduction in the exogenous GIR required to maintain euglycemia over the OGL period^6^. We also followed this strategy, where the exogenous GIR used was the average of the GIR that existed prior to ingestion of the OGL and that which existed at the end of the study. However, because plasma glucose rose unexpectedly in response to the OGL in our subjects, the accompanying increase in glucose utilization was accounted for by referring to the data of Hansen and colleagues^24^. In that study, it was shown that at steady state insulin levels, peripheral glucose utilization (PGU) in insulin resistant human subjects increases by 0.0425 mg/kg/min per 1 mg/dL rise in the plasma glucose level when PGU is 7.5 mg/kg/min and the plasma glucose level is 95 mg/dL. Because PGU was lower than this in our T2D subjects, this emendation was applied on a sliding scale, thereby resulting in the following calculation for SGE:

$$\begin{array}{l}{\mathrm{SGE}} = \left( {{\mathrm{GIR}}_{{\mathrm{avg}}} - {\mathrm{GIR}}_{t\,0 - 10}} \right) \cdot {\mathrm{kg}} \cdot {\mathrm{d}}t + \\ \left[ {0.0425 \cdot \frac{{{\mathrm{GIR}}_{{\mathrm{avg}}}}}{{7.5}} \cdot \left( {\frac{{{\mathrm{glucose}}_t \ + \ {\mathrm{glucose}}_{t + 10}}}{2} - 95} \right) \cdot {\mathrm{kg}} \cdot {\mathrm{d}}t} \right]\end{array}$$

Where GIR_avg_ equals the average of the GIRs that existed prior to and after intestinal absorption of the OGL (in mg/kg/min); GIR_*t*0–10_ is the average GIR over the 10-min period being calculated; kg is body mass in kg and d*t* is the time interval (10 mins). This calculation was made for every 10-min interval over the OGL-clamp absorption period and summed to provide a final value for SGE. SGU was finally determined by subtracting the final value for SGE from the amount of glucose ingested (75 g).

### Statistical analysis

Data were analyzed using repeated measures ANOVA and post-hoc analyses were made as appropriate using the Student–Newman–Keuls method. Data are summarized as mean ± sem, unless noted otherwise.

## Results

### Screening visit

#### Anthropometrics

Anthropometric data are presented in Table 1. Subjects who participated in the study included six obese people with type 2 diabetes, that performed aerobic exercise for 15 ± 2 weeks (AEX) and five subjects with T2D who remained sedentary for the same length of time (SED; 15 ± 1 weeks). VO_2_ max was similar in each group at baseline and subjects from both groups remained weight stable over the study’s duration. As opposed to SED, whose hemoglobin A1C did not change (*n* = 4 in SED for this measure), hemoglobin A1C was lower in AEX after the study (*p* < 0.05; *n* = 6), thereby indicating that the training program had a beneficial effect on whole-body glucose metabolism.

**Table 1:** Anthropometric characteristics of research participants

Group (gender)	Time	Age (years)	Height (cm)	Weight (kg)	BMI	VO2 max (mL/kg/min)	Hemoglobin A1C (%)
SED 2 m/3f	Pre	49 ± 5	172 ± 10	105 ± 14	36 ± 3	21.4 ± 3.3	7.1 ± 0.7
	Post	–	–	108 ± 14	36 ± 3	–	7.3 ± 1.1
AEX 2 m/4f	Pre	52 ± 6	165 ± 5	95 ± 7	35 ± 3	21.3 ± 4.9	7.5 ± 0.4
	Post	–	–	94 ± 7	35 ± 3	–	7.1 ± 0.2*

*m/f* male/female

Values are mean ± sd. *p < 0.05 compared to pre-intervention

#### Screen OGTT

Fasting plasma glucose was higher in AEX compared to SED (181 ± 5 vs 129 ± 7 mg/dL, respectively; *p* < 0.05). Two hours after ingestion of the 75 g OGTT, the plasma glucose level remained elevated but was not different between groups (321 ± 17 vs 254 ± 23 mg/dL, respectively; *p* = NS). Importantly, however, the change in plasma glucose in response to the 75 g oral glucose load was similar in each group (140 ± 10 and 125 ± 16 mg/dL in AEX and SED, respectively; *p* = NS), suggesting similar glucose tolerance. All subjects had a 2-h plasma glucose level >200 mg/dL. Plasma insulin levels were similar between groups at baseline (26 ± 4 and 30 ± 3 µU/mL in AEX and SED, respectively), and at the 120 min mark of the OGTT (74 ± 18 vs 110 ± 29 µU/mL, respectively; *p* = NS).

### Isoglycemic/hyperinsulinemic clamp

#### Pre-Intervention

##### Basal period

**Fig. 1:**
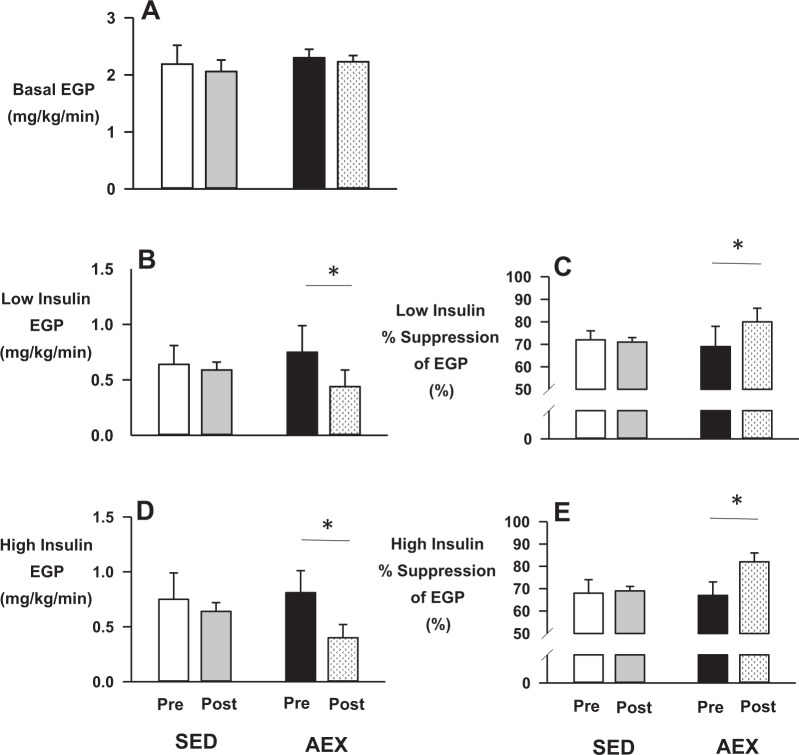
Metabolic responses to the ISO-Clamp studies. Endogenous glucose production (EGP) during the basal period (panel **a**) and during the low- and high-insulin infusion periods (panels **b** and **d**, respectively). Panels **c** and **e** are the respective percent suppression of EGP during each period. *p < 0.05

During the basal period of the pre-intervention isoglycemic/ hyperinsulinemic clamp, fasting plasma c-peptide and insulin concentrations were similar in AEX and SED, whereas plasma glucagon was elevated in SED (Table 2). This elevation in glucagon in SED, however, did not elevate EGP (Fig. 1a) or fasting plasma glucose concentrations (Table 2) compared to AEX. Levels of cortisol, NEFA and glycerol were also similar among groups (Table 2).

**Table 2:** Hormone and substrate levels during the isoglycemic/hyperinsulinemic clamp studies

			Time (min)											
Variable	Group	Time point	Basal period				Low- insulin period (20 mU/m^2^/min)				High- insulin period (40 mU/m^2^/min)			
			120	130	140	150	270	280	290	300	420	430	440	450
Plasma glucose (mg/dL)	SED	Pre	131 ± 21	131 ± 21	131 ± 21	131 ± 22	129 ± 21	130 ± 21	129 ± 21	130 ± 21	131 ± 22	131 ± 22	132 ± 22	131 ± 21
		Post	140 ± 22	141 ± 22	139 ± 22	138 ± 22	144 ± 24	143 ± 23	142 ± 23	141 ± 23	145 ± 27	144 ± 27	144 ± 27	142 ± 26
	AEX	Pre	168 ± 11	169 ± 11	167 ± 11	170 ± 12	164 ± 11	164 ± 10	164 ± 9	162 ± 9	158 ± 12	163 ± 13	161 ± 12	162 ± 12
		Post	167 ± 8	163 ± 9	163 ± 9	164 ± 9	165 ± 8	165 ± 8	166 ± 7	166 ± 7	162 ± 11	162 ± 9	162 ± 9	159 ± 10
Plasma insulin (µU/mL)	SED	Pre	26 ± 5	26 ± 4	26 ± 4	24 ± 4	51 ± 4	52 ± 4	52 ± 5	54 ± 5	91 ± 10	93 ± 11	98 ± 11	96 ± 10
		Post	23 ± 4	24 ± 4	23 ± 3	23 ± 4	49 ± 8	48 ± 7	49 ± 5	49 ± 7	84 ± 9	89 ± 11	86 ± 11	91 ± 11
	AEX	Pre	21 ± 4	21 ± 3	21 ± 3	20 ± 3	47 ± 6	44 ± 6	46 ± 6	47 ± 6	85 ± 18	89 ± 18	95 ± 18	96 ± 20
		Post	28 ± 6	27 ± 5	27 ± 5	27 ± 5	58 ± 10	57 ± 7*	59 ± 9*	56 ± 9*	79 ± 6	82 ± 7	82 ± 6	84 ± 7
Plasma C-peptide (ng/mL)	SED	Pre	3.3 ± 0.3	–	–	3.2 ± 0.4	2.6 ± 0.3	–	–	2.7 ± 0.3	2.3 ± 0.4	–	–	2.5 ± 0.5
		Post	2.8 ± 0.2	–	–	2.9 ± 0.2	2.7 ± 0.3	–	–	2.7 ± 0.3	2.5 ± 0.3	–	–	2.5 ± 0.2
	AEX	Pre	3.1 ± 0.4	–	–	2.9 ± 0.4	2.6 ± 0.3	–	–	2.8 ± 0.4	2.2 ± 0.4	–	–	2.3 ± 0.4
		Post	3.0 ± 0.4	–	–	2.9 ± 0.4	2.6 ± 0.4	–	–	2.6 ± 0.5	2.3 ± 0.5	–	–	2.3 ± 0.5
Plasma glucagon (pg/mL)	SED	Pre	67 ± 8	–	–	74 ± 12	61 ± 9	–	–	58 ± 4	41 ± 1	–	–	46 ± 4
		Post	46 ± 4*	–	–	46 ± 4*	37 ± 2*	–	–	35 ± 4*	27 ± 4	–	–	27 ± 3*
	AEX	Pre	45 ± 7†	–	–	45 ± 8†	40 ± 9	–	–	38 ± 9†	33 ± 8	–	–	36 ± 8
		Post	44 ± 5	–	–	42 ± 6	38 ± 7	–	–	36 ± 6	30 ± 7	–	–	29 ± 6
Plasma cortisol (µg/dL)	SED	Pre	15 ± 4	–	–	15 ± 4	13 ± 2	–	–	12 ± 1	12 ± 1	–	–	12 ± 1
		Post	15 ± 2	–	–	14 ± 2	13 ± 1	–	–	15 ± 1	14 ± 2	–	–	16 ± 2
	AEX	Pre	15 ± 3	–	–	13 ± 2	13 ± 3	–	–	12 ± 2	10 ± 1	–	–	9 ± 1
		Post	16 ± 2	–	–	15 ± 1	14 ± 1	–	–	13 ± 2	12 ± 1	–	–	14 ± 2*
Plasma NEFA (µmol/L)	SED	Pre	586 ± 68	–	–	590 ± 53	195 ± 47	–	–	197 ± 41	124 ± 17	–	–	153 ± 29
		Post	612 ± 44	–	–	580 ± 57	216 ± 42	–	–	208 ± 46	138 ± 23	–	–	120 ± 20*
	AEX	Pre	527 ± 76	–	–	520 ± 84	153 ± 28	–	–	142 ± 33	94 ± 21	–	–	89 ± 13†
		Post	360 ± 45*†	–	–	398 ± 66	129 ± 28	–	–	122 ± 22	68 ± 13†	–	–	68 ± 14
Plasma glycerol (µmol/L)	SED	Pre	87 ± 15	–	–	104 ± 11	58 ± 19	–	–	61 ± 16	36 ± 13	–	–	38 ± 11
		Post	119 ± 34	–	–	111 ± 32	65 ± 20	–	–	69 ± 25	65 ± 26*	–	–	57 ± 21
	AEX	Pre	102 ± 16			97 ± 17	53 ± 9			54 ± 8	40 ± 9			40 ± 6
		Post	74 ± 8			78 ± 8	46 ± 5			42 ± 7	37 ± 2			32 ± 3

Data are mean ± sem

*p < 0.05 for post versus pre values

†*p* < 0.05 for AEX compared to SED at the same time point

##### Low insulin period

In response to the IV infusion of insulin at 20 mU/m^2^/min, venous levels of the hormone were increased twofold in each group, while the level of both c-peptide and glucagon fell (Table 2). Despite this increase in plasma insulin, isoglycemia (Table 2) was maintained in both groups by IV infusion of dextrose (1.16 ± 0.05 and 1.35 ± 0.28 mg/kg/min in SED and AEX, respectively; *p* = NS). Endogenous glucose production (EGP) fell similarly in both SED and AEX (Fig. 1b). Peripheral glucose uptake (PGU) did not increase above what was observed for whole-body glucose turnover during the basal state in either group (1.81 ± 0.20 and 2.10 ± 0.37 mg/kg/min in SED and AEX, respectively), thereby demonstrating significant peripheral insulin resistance (Fig. 1f). Because of hyperinsulinemia, NEFA and glycerol in the plasma declined in both groups (Table 2).

##### High-insulin period

During the high-insulin infusion period, the IV-insulin levels were doubled again, resulting in c-peptide and glucagon falling further (Table 2). Again, plasma glucose (Table 2) was clamped in both groups by peripheral infusion of dextrose that averaged 2.45 ± 0.36 and 3.44 ± 0.81 mg/kg/min in SED and AEX, respectively (*p* = NS). EGP remained suppressed during the high-insulin period in both groups (Fig. 1d, e), as PGU rose in both groups above the level seen during the basal period (3.21 ± 0.58 and 4.25 ± 0.83 mg/kg/min in SED and AEX, respectively). Plasma glycerol and FFA continued to decline in each group from the low insulin infusion period.

#### Post-intervention

##### Basal period

During the basal period of the post-intervention isoglycemic/ hyperinsulinemic clamp, the plasma glucose, insulin and c-peptide levels within each group were similar to pre-intervention levels (Table 2). In addition, the elevated basal glucagon level that was observed in SED during the pre-intervention clamp was lower during the post-intervention study (*p* < 0.05 for each time point during post- compared to pre-intervention values in SED; Table 2). The levels of glucose, insulin, c-peptide, glucagon and cortisol were not different between SED and AEX during the basal period of their post-intervention metabolic study. During the basal period of the post-intervention metabolic study, EGP in SED and AEX remained unchanged compared to their respective pre-intervention values (Fig. 1a).

##### Low insulin period

During the low insulin infusion period of the post-intervention isoglycemic/ hyperinsulinemic study, the plasma glucose level was again clamped at a similar level in each group. As with the pre-intervention clamp studies, the insulin infusion doubled the hormone’s level in plasma, causing a further decline in both c-peptide and glucagon (Table 2). The glucose infusion rate required to maintain isoglycemia in SED (1.23 ± 0.13 mg/kg/min) was similar to what it was during the pre-intervention clamp (1.16 ± 0.05 mg/kg/min), whereas the glucose infusion rate in AEX increased from its pre-intervention value of 1.35 ± 0.28 mg/kg/min to 2.10 ± 0.32 mg/kg/min (*p* = 0.01). EGP was similar during the pre- and post-intervention clamps in SED during the low insulin infusion period (Fig. 1b). In AEX, however, both EGP and percent suppression of EGP changed favorably after the training period (Fig. 1b, c; *p* = 0.03 for each). In SED, PGU was unchanged from baseline during the low insulin infusion period of the post-intervention clamp (1.82 ± 0.20 mg/kg/min). PGU in AEX was increased during the post-intervention clamp compared with the pre-intervention clamp, but the difference was not statistically significant (2.54 ± 0.25 mg/kg/min).

##### High-Insulin Period:

During the high-insulin infusion, the post-intervention plasma glucose level remained similar between groups (Table 2). As with the pre-intervention clamp, the increase in circulating insulin concentrations further suppressed c-peptide and glucagon levels (Table 2). The glucose infusion rate required to maintain isoglycemia in SED (2.72 ± 0.22 mg/kg/min) was similar to the pre-intervention study (2.45 ± 0.36 mg/kg/min; *p* = NS), while the amount of glucose required to maintain isoglycemia in AEX increased from 3.44 ± 0.81 pre-exercise to 4.59 ± 0.89 mg/kg/min post-exercise (*p* < 0.01). EGP during high-insulin infusion was similar to the pre-intervention clamp in SED (Fig. 1d, e; *p* = NS), whereas EGP (*p* = 0.02) and the percent suppression of EGP (*p* = 0.01) were improved in AEX during the post-intervention clamp (Fig. 1d, e, **respectively**). During this period, PGU remained similar to the pre-intervention value in SED (3.36 ± 0.29 mg/kg/min), but increased to 5.01 ± 0.91 in AEX (*p* = 0.07).

### OGL-clamp

#### Pre-Intervention

##### Euglycemic/hyperinsulinemic lead-in period

**Fig. 2:**
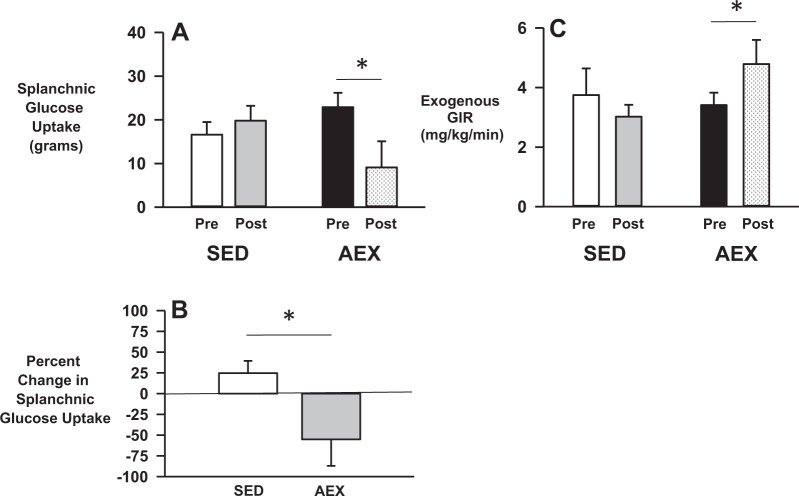
Metabolic responses to the OGL-Clamp studies. Splanchnic glucose uptake (**a**) in response to a 75 gram oral glucose load given to people with type 2 diabetes before (pre) and after (post) 15 weeks of aerobic exercise (AEX) or continued sedentary lifestyle (SED). Panel **b** is the pre-post percent change in splanchnic glucose uptake in each group. Panel **c** is the exogenous GIR immediately preceding ingestion of the oral glucose load. *p < 0.05

During the pre-intervention OGL-clamp’s lead-in period (−30 to 0 min), the IV-insulin levels were raised in both SED and AEX (Table 3). Meanwhile, the plasma glucose levels (Table 3) were clamped at euglycemia in both groups by a similar exogenous GIR in each group (Fig. 2c). Plasma c-peptide, glucagon, and cortisol were similar in each group and acetaminophen was negligible in the plasma. This hormonal milieu suppressed EGP to −0.03 ± 0.25 and 0.29 ± 0.15 mg/kg/min (*p* = NS) in SED and AEX, respectively, thereby making PGU and the exogenous GIR functionally the same.

**Table 3:** Hormone and substrate levels during the OGL-clamp studies

Variable	Time (min)												
	Group	Time point	Pre-OGL period				OGL period						
			−30	−20	−10	0	30	60	90	120	150	180	210
Plasma glucose (mg/dL)	SED	Pre	99 ± 2	97 ± 2	97 ± 2	98 ± 1	121 ± 10	139 ± 20	138 ± 22	119 ± 17	103 ± 7	89 ± 5	92 ± 5
		Post	96 ± 1	96 ± 1	94 ± 2	94 ± 2	128 ± 12	154 ± 18	156 ± 23	135 ± 24	112 ± 21	100 ± 10*	99 ± 2
	AEX	Pre	100 ± 2	99 ± 3	97 ± 4	97 ± 4	142 ± 7	160 ± 14	155 ± 14	121 ± 13	99 ± 3	94 ± 3	88 ± 3
		Post	98 ± 2	97 ± 3	97 ± 3	96 ± 3	130 ± 11	145 ± 11	143 ± 6	117 ± 10	102 ± 4	99 ± 2	99 ± 5
Plasma insulin (µU/mL)	SED	Pre	254 ± 28	281 ± 39	275 ± 35	254 ± 25	293 ± 55	310 ± 47	298 ± 41	299 ± 41	301 ± 28	299 ± 40	294 ± 34
		Post	268 ± 13	282 ± 17	271 ± 25	263 ± 9	284 ± 30	312 ± 41	301 ± 21	299 ± 25	290 ± 21	271 ± 15	291 ± 24
	AEX	Pre	267 ± 17	265 ± 27	269 ± 26	272 ± 18	316 ± 34	297 ± 37	327 ± 39	326 ± 43	295 ± 36	321 ± 42	330 ± 53
		Post	285 ± 23	281 ± 22	295 ± 26	287 ± 21	314 ± 32	298 ± 32	282 ± 34	307 ± 39	326 ± 36	318 ± 29	299 ± 26
Plasma C-peptide (ng/mL)	SED	Pre	1.8 ± 0.5	–	–	1.6 ± 0.5	–	3.6 ± 0.9	–	3.3 ± 1.0	–	2.4 ± 1.0	–
		Post	1.2 ± 0.2*	–	–	1.1 ± 0.3*	–	3.2 ± 0.6	–	2.8 ± 0.7	–	2.0 ± 0.9	–
	AEX	Pre	1.2 ± 0.2	–	–	1.1 ± 0.2	–	3.0 ± 0.8	–	2.6 ± 0.7	–	1.5 ± 0.3	–
		Post	1.2 ± 0.2	–	–	1.3 ± 0.2	–	2.7 ± 0.6	–	2.4 ± 0.6	–	1.6 ± 0.4	–
Plasma glucagon (pg/mL)	SED	Pre	45 ± 4	–	–	41 ± 5	–	45 ± 4	–	44 ± 4	–	42 ± 3	–
		Post	41 ± 7	–	–	36 ± 6	–	32 ± 3	–	33 ± 3	–	31 ± 4	–
	AEX	Pre	41 ± 7	–	–	42 ± 6	–	42 ± 6	–	38 ± 5	–	39 ± 6	–
		Post	33 ± 6	–	–	35 ± 7	–	37 ± 7	–	33 ± 8	–	30 ± 7	–
Plasma cortisol (µg/dL)	SED	Pre	–	–	–	14 ± 2	–	–	–	–	–	10 ± 2	–
		Post	–	–	–	14 ± 2	–	–	–	–	–	10 ± 2	–
	AEX	Pre	–	–	–	15 ± 2	–	–	–	–	–	12 ± 2	–
		Post	–	–	–	16 ± 2	–	–	–	–	–	16 ± 1†	–
Plasma acetaminophen (µg/mL)	SED	Pre	0 ± 0	–	–	0 ± 0	10 ± 1	15 ± 1	19 ± 2	19 ± 2	17 ± 2	14 ± 1	13 ± 2
		Post	0 ± 0	–	–	0 ± 0	13 ± 2	17 ± 1	21 ± 2	20 ± 2	15 ± 2	13 ± 1	12 ± 1
	AEX	Pre	0 ± 0	–	–	0 ± 0	11 ± 2	19 ± 2	23 ± 2	19 ± 1	16 ± 1	14 ± 2	11 ± 2
		Post	0 ± 0	–	–	0 ± 0	12 ± 1	18 ± 2	20 ± 1	20 ± 1	16 ± 1	14 ± 1	11 ± 2
Plasma NEFA (µmol/L)	SED	Pre	112 ± 10	–	–	–	–	–	–	–	–	78 ± 13	–
		Post	127 ± 10	–	–	–	–	–	–	–	–	91 ± 13	–
	AEX	Pre	77 ± 5†	–	–	–	–	–	–	–	–	79 ± 12	–
		Post	72 ± 9†	–	–	–	–	–	–	–	–	67 ± 14	–
Plasma glycerol (µmol/L)	SED	Pre	40 ± 7	–	–	–	–	–	–	–	–	32 ± 5	–
		Post	53 ± 15	–	–	–	–	–	–	–	–	44 ± 11	–
	AEX	Pre	39 ± 5	–	–	–	–	–	–	–	–	34 ± 5	–
		Post	33 ± 5	–	–	–	–	–	–	–	–	35 ± 6	–

Data are mean ± sem

*p < 0.05 for post versus pre values

†*p* < 0.05 for AEX compared to SED at the same time point

##### OGL-clamp period

After consuming the 75-g glucose load, the plasma insulin level remained similar in each group, indicating that endogenous insulin secretion was markedly inhibited by the high-insulin infusion rate. On the other hand, the levels of glucose and acetaminophen in the plasma rose similarly in both SED and AEX (Table 3), before glucose returned to pre-OGL levels. Interestingly, despite the prevailing hyperinsulinemia, plasma c-peptide more than doubled in response to the OGL, after which it waned over time; this, however, had only a small (~10–15%) effect on plasma insulin levels and was not different between groups. The OGL likely had no effect on pre-intervention plasma glucagon concentrations in either group because of the pre-existing hyperinsulinemia (Table 3). SGU was similar in SED and AEX during the pre-intervention clamp (Fig. 2; *p* = NS).

#### Post intervention

##### Euglycemic/hyperinsulinemic Lead-In Period:

As was the case during the pre-intervention clamp, insulin levels were similar in SED and AEX during the lead-in period of the post-intervention OGL-clamp (Table 3). Likewise, there were no differences between groups in c-peptide or glucagon between groups and acetaminophen was undetectable. The GIR required to maintain euglycemia was unchanged in SED, but increased in AEX (Fig. 2c; *p* = 0.01). This hormonal milieu completely suppressed EGP (0.02 ± 0.19 and −0.13 ± 0.20 mg/kg/min in SED and AEX, respectively), thereby making PGU tantamount to the GIR.

##### OGL-clamp period

Despite slightly higher and lower plasma glucose levels in SED and AEX, respectively, the glucose AUC in response to the post-intervention OGL was similar in all four groups (Table 3). Likewise, acetaminophen from the OGL rose and fell in concert in both groups, thereby indicating no difference in the intestinal absorption of glucose in response to exercise training (Table 3). As was the case during the pre-intervention OGL-clamp, ingestion of the OGL increased c-peptide similarly in both groups but did not impact glucagon or cortisol (Table 3). In response, SGU was unchanged in SED, but markedly reduced in AEX (Fig. 2a; *p* = 0.04); a 25 ± 15% increase and 55 ± 32% decrease in the respective groups (Fig. 2b; *p* = 0.04).

## Discussion

Obesity-associated T2D is characterized by insulin resistance and the dysfunction of several glucoregulatory organs including skeletal muscle and liver. Importantly however, exercise training improves insulin resistance in these tissues, thereby making it a cornerstone in the treatment of the disease. As a considerable amount of research continues to focus on the mechanistic basis of exercise-induced improvements in muscle insulin sensitivity, much less is known about how exercise affects liver function in T2D, which is unfortunate, given the central role the liver plays in the regulation of blood glucose homeostasis. With this in mind, we investigated the effect of aerobic exercise training on hepatic glucose metabolism, during both the fasted- and fed-states, in patients with T2D.

Endogenous glucose production (EGP) is elevated in human patients with T2D, and the suppression of EGP in response to hyperinsulinemia is diminished^25,26^. At the cellular level, this is accounted for by elevated rates of both gluconeogenesis and glycogenolysis which, in turn, increase the flux of substrate through glucose-6-phosphatase (G6Pase) and glucose production. Our data demonstrate that 15 weeks of aerobic exercise training improved EGP suppression over a broad range of physiological hyperinsulinemia, thereby contributing to improved glycemic regulation. Although we did not investigate the mechanisms responsible for this improvement, it likely occurs as a function of increased activity of glucokinase relative to glucose-6-phosphatase. Activity of these enzymes is known to be unbalanced in T2D, such that it causes elevated EGP in this population^27,28^. Because each enzyme’s activity is regulated by insulin^29^, improved hepatic sensitivity to the hormone after exercise training would be expected to lower EGP during hyperinsulinemia.

An unanticipated finding was that exercise training reduced SGU in response to a 75 g oral glucose load. Even more surprisingly though, this occurred even with gains in hepatic insulin sensitivity. While this finding could be interpreted as unfavorable, it occurred in association with a reduction in hemoglobin A1C over the 15-week intervention period, thereby indicating a positive net effect of exercise training on glucoregulation over the same period.

Because catheterizing blood vessels into and out of the liver is prohibitively invasive, hepatic glucose uptake cannot be directly measured in humans. A surrogate for the measurement of SGU, however, is the OGL-clamp. This method has been validated in humans^6^ and, along with more mechanistic studies in animals, its use has shown that the liver plays an important role in postprandial glucose metabolism. Not only does the liver lower its own glucose production in response to a meal, it also takes up approximately one-third of ingested carbohydrate, storing most of it as glycogen for later use during fasting^6,7,9^. A number of metabolic cues facilitate maximal postprandial hepatic glucose uptake, including hyperinsulinemia, hyperglycemia and a negative arterial-hepatic portal vein glucose gradient^30–34^. Because SGU is impaired in subjects with T2D^5,10–12^, we hypothesized that exercise-induced improvements in hepatic insulin sensitivity would increase it. Interestingly however, the opposite actually occurred. Despite exhibiting improved hepatic insulin sensitivity during the ISO-clamp, a marked reduction in SGU was observed during the OGL-clamp after exercise training; a finding that could be interpreted as diminished hepatic function. However, Maehlum et al.^35^, observed a similar result in healthy humans after they exercised exhaustively just prior to an oral glucose load, and when the exercise happened 14–15 h before the OGL. Likewise, in canine studies, Wasserman and colleagues found that intraduodenal glucose infusion immediately after an exercise bout increased splanchnic glucose output due to accelerated intestinal absorption of the sugar^36,37^. The observation that healthy humans and animals- with intact glucose metabolism- show diminished SGU both immediately and 14–15 h after exercise, suggests that the lower SGU in our T2D subjects after exercise training is not maladaptive. Illustrating this is the fact that insulin-induced muscle glucose uptake more than accounted for the reduction in SGU. In AEX, the average PGU during the OGL-clamp was 3.8 mg/kg/min prior to the intervention and 5.1 after it. This makes the increase in peripheral glucose uptake after exercise ~22 g (over 180 min), which is 58% higher than the reduction in SGU (13.8 g) and may represent a repartitioning of glucose disposal away from the splanchnic bed in favor of replenishing muscle glycogen.

At this time, the mechanism that reduced SGU after exercise training remains unclear. Based on the acetaminophen data, a rise in intestinal glucose absorption does not appear to explain the reduction in SGU. On the other hand, Knudsen and colleagues^38^, showed that liver glycogen content is increased in muscle IL-6 knockout mice at rest and after exercise. This raises the possibility that inter-organ crosstalk, facilitated by exercise-induced myokines such as IL-6, could orchestrate the subsequent reduction in SGU during the postprandial state. Future studies will be needed to verify this hypothesis and will also be required to differentiate between the effect of an acute bout of exercise on hepatic glucose metabolism compared to chronic training.

Despite the plasma glucose concentration being similar in both groups before and after the intervention period, it rose invariably above basal (95 mg/dL) during the first ~60–90 min after the 75 g OGL, despite hyperinsulinemia of ~300 µU/mL. We are not aware of any similar reports of hyperglycemia using the OGL-clamp method, but this demonstrates the significant insulin resistance of our T2D cohort, which was likely exacerbated by the discontinuance of their diabetes-related medications for the previous five days. On the one hand, the increase in plasma glucose more closely mimics the rise seen in response to oral glucose ingestion^39^. On the other hand, we were required to account for the increase in the plasma glucose level when calculating splanchnic glucose escape. To do so, we utilized the work of Hansen et al.^24^, who showed that at steady state insulin levels, the relationship between PGU and the rise in plasma glucose is linear and is not impacted in vivo by insulin resistance^24,40^. All told, the largest proportion (>80%) of calculated splanchnic glucose escape was still accounted for by the AUC of the fall in the GIR during the OGL period. Likewise, because the AUC for plasma glucose responses to the OGL were similar in both groups regardless of time, the GIR-derived portion of the calculation accounted for the entirety of the increase in splanchnic glucose escape (and thus the lowering of SGU) after exercise training. In the unlikely event that we underestimated splanchnic glucose escape because exercise training enhanced the relationship between the plasma glucose level and muscle glucose uptake at steady state insulin levels of ~300 µU/mL, it would have led to an underestimation of peripheral glucose uptake and splanchnic glucose output after training. In turn, it would mean that the reduction in SGU after exercise training is even greater than what we report.

In summary, the current results further demonstrate that in addition to markedly improving muscle insulin resistance in obese human subjects with T2D, aerobic exercise training also improves insulin-mediated suppression of EGP. Despite this improvement in hepatic insulin sensitivity, however, SGU during the postprandial state is diminished after exercise training. We hypothesize that this reduction marks a diversion of ingested glucose away from the liver in favor of skeletal muscle glycogen repletion. Future studies will be required to determine the mechanistic basis for this repartitioning, as it may involve crosstalk between skeletal muscle and the liver.
